# Regulation of red cell life-span, erythropoiesis, senescence, and clearance

**DOI:** 10.3389/fphys.2014.00269

**Published:** 2014-07-18

**Authors:** Lars Kaestner, Anna Bogdanova

**Affiliations:** ^1^Research Center for Molecular Imaging and Screening, Medical School, Institute for Molecular Cell Biology, Saarland UniversityHomburg/Saar, Germany; ^2^Vetsuisse Faculty, and the Zurich Center for Integrative Human Physiology, Institute of Veterinary Physiology, University of ZurichZurich, Switzerland

**Keywords:** erythrocyte, erythropoiesis, senescence, clearance, blood storage, neocytolysis, vesiculation

The number of red blood cells (RBCs) and their properties are optimized by nature for most efficient oxygen delivery from the lungs to hypoxic periphery. Changes in metabolic requirements or environmental oxygen availability quickly translate to modulation of the RBC number, blood rheology, oxygen affinity of hemoglobin, and even of vascular tone. Inability to match the changes in oxygen demand may be fatal and requires therapeutic intervention. The recent advances in the ongoing intensive investigations of the mechanisms in control of regulation of erythropoiesis, RBC maturation and aging, as well as the processes involved in recognition of senescent RBCs and their clearance make up the present volume.

It all starts from within the mesoderm, the fetal liver and from the adult bone marrow where primitive or definitive erythropoiesis takes place (Palis, 2014). Facilitated RBC production may be induced promptly “on demand” in extended oxygen requirements upon ascent to the high altitude and quickly reversed when extra RBC mass is without benefit any more (Risso et al., 2014). Exercise and professional sport increase RBC turnover and maximize oxygen delivery to the tissues (Mairbäurl, 2013). Maturation and aging of RBCs is accompanied by multiple processes occurring at various rates driving the circulating RBCs from adolescence to senescence within approximately 120 days (Lew and Tiffert, 2013; Lutz and Bogdanova, 2013). The resulting “markers of senescence” are recognized by the macrophages and clearance of RBCs is promptly initiated (de Back et al., 2014). Premature clearance is a hallmark of various disorders associated with anemia. In each case one or multiple markers of senescence appear prematurely. Those include excessive oxidative stress (Mohanty et al., 2014), excessive cation leak with the following dehydration (Wang et al., 2014), decrease in RBC size and loss of RBC membrane through vesiculation (Alaarg et al., 2013), metabolic abnormalities (Vives-Corrons et al., 2013), or following auto-immune diseases (Lutz and Bogdanova, 2013). Blood storage damages RBCs facilitating aging. As a result clearance of transfused cells is dramatically facilitated (Bosman, 2013; Flatt et al., 2014).

The present compilation does not only give an overview of the variety of opinions reflecting the current understanding of the mechanisms of erythropoiesis, aging, and clearance of RBCs. We hope that it also provides the base for future lively discussions of the up-standing problems in this rapidly developing research area.

## Conflict of interest statement

The authors declare that the research was conducted in the absence of any commercial or financial relationships that could be construed as a potential conflict of interest.
